# Medical certification of incapacity in guardianship applications: conceptualising capacity

**DOI:** 10.1192/pb.bp.113.044719

**Published:** 2016-02

**Authors:** Tom C. Russ, Alison Thomson, Donald Lyons

**Affiliations:** 1 University of Edinburgh, UK; 2 Mental Welfare Commission for Scotland, Edinburgh, UK

## Abstract

**Aims and method** To examine how capacity is recorded in practice and compare this with the statutory definition, medical reports accompanying a random 10% sample (183 applications; 360 reports) of guardianship applications granted in 2011-2012 were examined.

**Results** Clinicians did not explicitly use the statutory definition of capacity in 47.5% of reports. Over half of applications (56.4%) did not explicitly link the powers sought with the patient's vulnerabilities; such a link was less common in older adults (*P* = 0.0175).

**Clinical implications** Guardianship orders can justify deprivation of liberty. Therefore it is important that such cases involve a thorough assessment of the person and that due process is followed, including adherence to the statutory definition of capacity. Practice could be improved by altering the paperwork required of medical practitioners, in line with mental health legislation. In addition, these findings should inform current legislation reform.

Mental capacity and consent to treatment are central to the practice of psychiatry and, indeed, all branches of healthcare. Fundamental to the process of making decisions on behalf of people who lack capacity is the definition of capacity used. The problem of numerous, slightly different definitions has been solved – in theory – by statutory definitions in Scotland (by the Adults with Incapacity (Scotland) Act 2000 (AWI)) and subsequently in England and Wales (by the Mental Capacity Act 2005). In AWI, incapacity is defined as being ‘incapable of acting; or making decisions; or communicating decisions; or understanding decisions; or retaining the memory of decisions’ (Section 1(6)). The General Medical Council (GMC) explicitly instructs doctors assessing capacity to use the statutory definition of the jurisdiction in which they work.^1^ The legal framework in Scotland, England and Wales involves a presumption in favour of capacity and an acknowledgement that capacity is decision specific.

Following cases such as *HL v United Kingdom* (the ‘Bournewood’ case),^2^ deprivation of liberty safeguards have been introduced in England and Wales to provide lawful justification of deprivation of liberty when this is necessary. No equivalent safeguards exist in Scotland, where it is likely that a court will appoint a guardian. Guardians can have welfare powers, financial powers or both, and can make decisions in these areas on behalf of the person lacking capacity. Thus, guardianship is a ‘procedure prescribed by law’, as required for legal justification of deprivation of liberty under the European Convention on Human Rights 1953 (ECHR). Article 5 of the ECHR (and the UK Human Rights Act 1998 which is based on it) allows deprivation of liberty only in certain specific circumstances; in a medical context, Section 1(e) is the most relevant: ‘No one shall be deprived of his liberty save … the lawful detention of persons for the prevention of the spreading of infectious diseases, of persons of unsound mind, alcoholics or drug addicts or vagrants’.

It is unknown how clinicians conceptualise capacity in practice, whether they record their judgement in line with the statutory definition, and whether they link the patient's capacity and specific vulnerabilities with the powers sought by the guardian. Therefore we present an analysis of medical reports accompanying applications for guardianship and discuss the definition of capacity used in the context of the powers requested.

## Method

Application for guardianship under AWI is made, either by private individuals or by the local authority, to the Sheriff Court and the application is accompanied by two medical certificates (see online supplement to this paper) and, where welfare powers are sought, a mental health officer's report. The core of the medical certificates consists of four questions regarding: the clinician's findings, the likely duration of the incapacity, communication, and consultation with relatives, carers or others having an interest in, or knowledge of, the person concerned.

The Mental Welfare Commission for Scotland is a statutory body which works to promote the welfare and safeguard the rights of individuals with mental illness, intellectual disability and related conditions. All applicants for guardianship orders including welfare powers are required to send a copy of the paperwork to the Commission. Of the guardianship orders granted between April 2011 and March 2012, 42.7% requested only welfare powers, 48.5% requested both welfare and financial powers, and 8.8% requested only financial powers.^3^ Correspondence received by the Mental Welfare Commission is scanned and stored electronically on a database, indexed by the patient's unique identifier. Data for all guardianship orders granted during the year 2011-2012 of which the Mental Welfare Commission was notified were extracted. A computer-generated random number was used to select a 10% sample of these applications and the scanned images of both medical reports accompanying these applications were examined.

Details of the adult's age, diagnosis and the powers sought under the guardianship order were recorded. The definition of capacity used was summarised and it was noted whether or not the statutory definition was used. Applications for guardianship will very carefully outline the powers requested for the individual, reflecting the decision-specific nature of capacity. Any link made between the powers sought and the individual's capacity and vulnerability was examined along with reference to the principles of AWI and whether the two reports were congruent.

The characteristics of the sample and all applications were compared with χ^2^-tests for categorical variables and *t*-tests for continuous variables. All analyses used R for Windows version 2.15.2 (http://cran.r-project.org/bin/windows/base/old/2.15.2/). In a supplementary analysis, reports for individuals aged 65 and over were compared with those for younger individuals.

## Results

A total of 1821 welfare guardianship orders were granted during the study period and reported to the Mental Welfare Commission; a random sample of 183 applications (360 reports – some applications were accompanied by a single medical report) were included in the study. Characteristics of the sample and the total guardianship orders granted are shown in Table 1. There were no differences in the distribution of characteristics in the sample compared with all applications. More than half were for women (59.6%) and there was a wide spread of ages, although 54.1% of applications related to an adult over the age of 65 years. The majority of applications were made by private individuals (74.3%) and more than half requested welfare and financial powers (57.4%). A substantial proportion of applications (45.4%) requested an indefinite duration of guardianship. Dementia was the most common condition causing the incapacity and, with intellectual disability (the next most common), constituted 84.2% of applications. Applications from all health board areas were examined, with the exception of Shetland and the Western Isles which had very small numbers of applications granted in the study period, none of which were randomly selected for inclusion.

**Table 1:** Characteristics of the sample

	Random sample *n* = 183	All applications *n* = 1821	*P*
Female, *n* (%)	109 (59.6)	1027 (56.4)	0.456

Age at approval, years: mean (s.d.)	61.6 (25.5)	59.6 (26.4)	0.335

Age groups, *n* (%)			
<18	15 (8.2)	154 (8.5)	0.380
18–24	12 (6.6)	196 (10.8)	
25–44	22 (12.0)	234(12.9)	
45–64	35 (19.1)	277 (15.2)	
65–84	62 (33.9)	640 (35.1)	
85+	37 (20.2)	320 (17.6)	

*Applications, n* (%)			
Private applications	136 (74.3)	1352 (74.2)	1
Of indefinite duration	83 (45.4)	824(45.2)	1
Powers requested			
Welfare powers	78 (42.6)	808 (44.4)	0.591
Welfare and financial powers	105 (57.4)	1013 (55.6)	

Diagnosis, *n* (%)			
Dementia	88 (48.1)	921 (50.6)	0.115
Intellectual disability	66 (36.1)	681 (37.4)	
Alcohol-related brain damage	12 (6.6)	57 (3.1)	
Brain injury	7 (3.8)	91 (5.0)	
Other^a^	10 (5.5)	71 (3.9)	

Health board region, *n* (%)			
Ayrshire and Arran	14 (7.7)	132 (7.2)	0.970
Borders	1 (0.5)	23 (1.3)	
Dumfries and Galloway	3 (1.6)	43 (2.4)	
Fife	12 (6.6)	141 (7.7)	
Forth Valley	5 (2.7)	79 (4.3)	
Grampian	18 (9.8)	183 (10.0)	
Greater Glasgow and Clyde	50 (27.3)	471 (25.9)	
Highland	14 (7.7)	134 (7.4)	
Lanarkshire	26 (14.2)	235 (12.9)	
Lothian	16 (8.7)	180 (9.9)	
Orkney	1 (0.5)	7 (0.4)	
Shetland	0 (0.0)	3 (0.2)	
Tayside	23 (12.6)	182 (10.0)	
Western Isles (Eilean Siar)	0 (0.0)	8 (0.4)	

a. Includes inability to communicate owing to a physical disorder, mental illness, personality disorder and other (not specified).

There was disagreement between the two medical reports for 26 individuals (14.1%), either in the record of powers requested or the diagnosis. Eleven applications (6.0%) had either only one report (as can be the case for renewal of guardianship) or had pages missing from the electronic record as a result of clerical error; there is no reason to suspect bias as a result of these missing data. The powers requested were blank on one report in three applications and once for both reports.

### Definition of capacity

Clinicians did not explicitly use the statutory definition of capacity in 171 reports (47.5%). Of the 182 reports which did use a definition of capacity in line with AWI (7 reports were missing the relevant pages), the vast majority (127; 35.3% of total) referred to making decisions either as the sole basis for incapacity (*n* = 106) or in combination with other aspects of the definition (*n* = 21). Thirteen reports (3.6%) included a blanket statement referring to the entire statutory definition, on occasions quoting verbatim from AWI. A number of reports were couched in terms of insight into the person's illness or even referred to the ‘significantly impaired decision making ability’ required for compulsory treatment under the Mental Health (Care & Treatment) (Scotland) Act 2003.

A total of 188 reports concerned an adult aged 65 years or older (excluding 7 reports which were missing relevant information), of which only 10 (5.3%) were not associated with a diagnosis of dementia or cognitive impairment (these applications concerned people with schizophrenia or intellectual disability). Of these reports concerning older adults, 88 (46.8%) explicitly used the statutory definition of capacity compared with 96/165 (58.2%) reports concerning adults under 65 years (χ^2^ = 4.1, *P* = 0.0426).

### Linking powers sought with capacity or vulnerability

The powers sought in the guardianship application were explicitly linked with the adult's capacity and/or vulnerability in 157 reports (43.6%) – 92/165 concerning adults under 65 years and 65/188 concerning adults aged 65 or older (55.8% *v*. 34.6%; χ^2^ = 5.7, *P* = 0.0175). Some reports provided explicit examples – sometimes extensive lists – of areas where the individual lacked capacity and highlighted the risks if welfare or financial powers were not in place under a guardianship order. In contrast, other reports – where there was any link made between capacity and the powers sought – had vague statements along the lines of ‘I believe [the adult] is unable to make decisions regarding matters of financial or personal welfare’.

## Discussion

Almost half of all medical reports accompanying guardianship orders in the present sample did not use or refer to the statutory definition of capacity and, of those that did, most only referred to the ability to make decisions, rather than the fuller definition in AWI. There was disagreement between the medical reports in approximately 14% of applications. Fewer than half of the medical reports were explicit in linking the powers sought with the adult's capacity or vulnerabilities. Applications regarding older adults, who may be subjected to the greatest deprivation of liberty, were even less likely to use the statutory definition of capacity or link the powers and the individual's vulnerability.

### Capacity and deprivation of liberty

As mentioned above, guardianship is a procedure prescribed by law – as required by the ECHR – to authorise deprivation of liberty in non-hospital care settings. In this context, it is similar in status to the Deprivation of Liberty Safeguards in England and Wales. Since depriving an individual of their liberty is such a serious matter, it is essential that the capacity assessment at the core of a guardianship application is conducted in line with the statutory definition laid down in AWI. However, despite the recognition that capacity is decision specific, there were 26 applications (14.1%) included in this study where the powers requested according to the two medical reports or the diagnosis did not agree, suggesting that at least one of the assessing doctors was not clear what powers were being requested in the guardianship order or even about the patient's condition.

In England and Wales the requirements for the need for the Deprivation of Liberty Safeguards are interpreted inconsistently and their use is patchy.^4^ Also, the interface with mental health legislation is problematic.^5^ Similarly in Scotland, both the use of guardianship and the mental health act pose problems of interpretation and implementation.^6^

### Legislative framework

In Scotland there was recently a consultation gathering opinion from all stakeholders as to how deprivation of liberty should be identified and managed in legislation.^7^ This consultation paper (and the Scottish Law Commission's final report^8^) contains detailed discussion of the legal situation in Scotland, the rest of the UK and elsewhere concerning people who lack capacity. In the *Muldoon* case Sheriff Baird was of the opinion, following the *Bournewood* judgments, that the least restrictive option regarding a person who lacked capacity but who was compliant was guardianship.^9^ Subsequent Scottish legislation (Adult Support and Protection (Scotland) Act 2007) has attempted to clarify how local authorities deal with adults with incapacity but the situation remains unclear – centring around the question of what constitutes deprivation of liberty. The Scottish Law Commission consultation paper^7^ is the start of a process asking questions about whether there needs to be a change in legislation regarding this group of patients and, if so, what form this change needs to take. Indeed, it is likely that all systems will have to adapt to provide a more appropriate response for this vulnerable group of people. However, the implications of requiring guardianship in all cases where an individual lacks capacity would be substantial, particularly considering projected demographic changes.

### Are things improving?

Shortly after AWI was introduced, the Mental Welfare Commission for Scotland carried out a similar analysis of 167 medical certificates accompanying applications for welfare guardianship between April 2002 and February 2003.^10^ This analysis found that 49% of applications drew attention to an individual's vulnerability rather than simply stating that they lacked capacity, higher than the 43.6% found in the present study. The two medical reports were found to disagree in 23% of cases, suggesting that this aspect of guardianship applications has improved. However, we can see that over a decade since the previous analysis, capacity assessment for guardianship applications has not become markedly better in terms of adherence to the statutory framework.

### Implications of this study

In the forms examined, detailed descriptions of an individual's capacity and specific vulnerabilities linked with the powers sought in the application were not universally recorded. Indeed, the forms completed by medical practitioners to accompany guardianship applications (see the online supplement to this paper) do not encourage this. In contrast to the extensive paperwork completed to detain someone under the Mental Health (Care and Treatment) (Scotland) Act 2003, there is relatively little information required from doctors completing the forms – it is possible to complete the paperwork correctly while giving very little information. Admittedly, these often accompany an extensive report by a mental health officer but the disparity is still striking. Revised forms which require more relevant details to be collected would provide more robust justification for consequent deprivation of liberty. A revised form for medical recommendations should require individual, separate statements about the adult's ability to act, make decisions, communicate decisions, understand decisions and retain the memory of decisions. Furthermore, the findings of the present study regarding medical certification of incapacity should inform the Scottish Government's current review of AWI.
